# Neural Network Training Acceleration With RRAM-Based Hybrid Synapses

**DOI:** 10.3389/fnins.2021.690418

**Published:** 2021-06-24

**Authors:** Wooseok Choi, Myonghoon Kwak, Seyoung Kim, Hyunsang Hwang

**Affiliations:** Department of Materials Science and Engineering, Pohang University of Science and Technology, Pohang, South Korea

**Keywords:** hardware neural networks, online training, resistive memory, hybrid synapse, crossbar array

## Abstract

Hardware neural network (HNN) based on analog synapse array excels in accelerating parallel computations. To implement an energy-efficient HNN with high accuracy, high-precision synaptic devices and fully-parallel array operations are essential. However, existing resistive memory (RRAM) devices can represent only a finite number of conductance states. Recently, there have been attempts to compensate device nonidealities using multiple devices per weight. While there is a benefit, it is difficult to apply the existing parallel updating scheme to the synaptic units, which significantly increases updating process’s cost in terms of computation speed, energy, and complexity. Here, we propose an RRAM-based hybrid synaptic unit consisting of a “big” synapse and a “small” synapse, and a related training method. Unlike previous attempts, array-wise fully-parallel learning is possible with our proposed architecture with a simple array selection logic. To experimentally verify the hybrid synapse, we exploit Mo/TiO_x_ RRAM, which shows promising synaptic properties and areal dependency of conductance precision. By realizing the intrinsic gain via proportionally scaled device area, we show that the big and small synapse can be implemented at the device-level without modifications to the operational scheme. Through neural network simulations, we confirm that RRAM-based hybrid synapse with the proposed learning method achieves maximum accuracy of 97 %, comparable to floating-point implementation (97.92%) of the software even with only 50 conductance states in each device. Our results promise training efficiency and inference accuracy by using existing RRAM devices.

## Introduction

Artificial intelligence (AI) technology is becoming increasingly advanced and widespread in real-world applications, such as computer vision, natural language recognition, healthcare, and pattern classification (Ghahramani, 2015; Mnih et al., 2015; Silver et al., 2016; Guo et al., 2020; McKinney et al., 2020). Advances in AI technology have been achieved through the unprecedented success of deep-learning algorithms. However, based on the von Neumann architecture, conventional digital computers cannot withstand the ever-increasing sizes and complexities of neural networks and tasks, thereby facing barriers in terms of energy efficiency (Merkel et al., 2016; Yan et al., 2019; Ankit et al., 2020). This has necessitated the development of brain-inspired neuromorphic computing, e.g., hardware neural networks (HNNs). In particular, resistive memory (RRAM) is considered a strong candidate for synaptic primitives capable of storing multilevel weights as conductance values (Woo et al., 2016; Yu et al., 2016; Wu et al., 2019; Yin et al., 2020). Especially in an RRAM array, fully-parallel array operations provide the excellent potential to accelerate neural network computations. However, as existing RRAMs can only represent a finite number of conductance states, it poses a significant challenge to achieving high accuracy of HNNs during online training (Li et al., 2015; Gokmen and Vlasov, 2016; Kim et al., 2017; Mohanty et al., 2017; Nandakumar et al., 2020).

To store a higher number of bits per weight, several studies have used multiple cells for a synapse in analog neuromorphic systems (Agarwal et al., 2017; Song et al., 2017; Boybat et al., 2018; Liao et al., 2018; Hsieh et al., 2019; Zhu et al., 2019). While there is a benefit, the synaptic unit architecture cannot adopt the conventional parallel updating scheme since multiple devices operate one synapse. The system must determine the device to be updated for each synaptic unit and calculate the weight updates’ corresponding amounts. As a result, the synaptic unit architecture’s update process requires additional expenses in terms of time and energy. In positional number systems, carry operations must be performed between the combined devices in every synaptic unit (Agarwal et al., 2017; Song et al., 2017), which is not compatible with the parallel updating scheme. Liao et al. proposed a synaptic unit with sign-based stochastic gradient descent training to implement the parallel updating (Liao et al., 2018). However, ignoring the magnitude information of the weight updates decreases the classification accuracy. Thus, for fast and accurate HNN learning, it is crucial to be able to train the synaptic unit architecture using the parallel update method without losing the amount of the feedback information.

Therefore, we propose a hybrid synaptic unit using Mo/TiO_x_ RRAMs with a cooperative training method that can accelerate the learning of neural networks with increased precision of the synaptic weight. The remainder of this paper is organized as follows. (1) We explain the importance of high precision of the synaptic element and the parallel updating scheme, which are essential for accelerating neural network training with high accuracy. (2) We present the hybrid synaptic unit consisting of “big” and “small” synapses. We also present the training method to simplify the updating process by separating the role of each synapse in the unit. We train the HNN in two phases wherein, first, a dynamic-tuning phase that only updates the big synapses is followed by a fine-tuning phase that only updates the small synapses in detail. Hence, the HNN can accelerate learning process by using a parallel updating scheme to the target array with simple array selection logic. (3) To implement the hybrid synapse experimentally, we exploit Mo/TiO_x_ RRAM that exhibits promising synaptic properties and the areal dependency of the conductance precision. By realizing the intrinsic gain via proportionally scaled device area, we show that the big and small synapse can be implemented at the device-level without modifications to the operational scheme. (4) By considering realistic device parameters, we conduct neural network simulations to confirm the feasibility of the proposed method. We also analyze the optimal gain ratio between the synapses to achieve the highest accuracy. The results demonstrate that hybrid synapse-based HNN with the proposed learning method significantly improves accuracy for handwritten digit datasets, which is 99.66% for training and 97% for the tests. We believe that this work is a meaningful step toward a high-performance RRAM-based neuromorphic system using existing RRAM devices.

## Hardware Neural Network

### Synaptic Device

**FIGURE 1:**
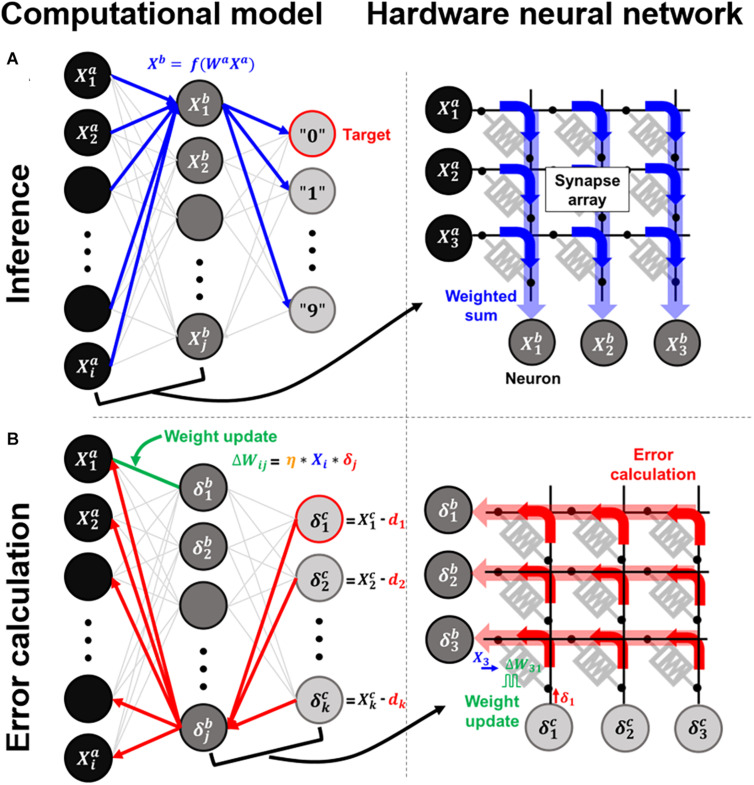
Computational models and arrays of hardware neural networks for the **(A)** inference process and **(B)** error calculation process.

The working principle of an HNN is based on parallel signal propagations in crossbar array architecture. For the synaptic weights (*W*_ij_), the conductance values of the resistive device are the weights indicating the strength of the synaptic connection. Herein, a synapse $\left(G_{\mathrm{ij}}^{+}-G_{\mathrm{ij}}^{-}\right)$ typically consists of two devices, where *G*^+^ and *G*^−^ represent the conductance states of the positive and negative devices, and the subscripts i and j are the crossbar array indexes. After the pre-neurons express voltage signals, these signals are naturally multiplied by the conductance values of the synapses using Ohm’s law. Thus, the signals from the pre-neurons are computed in the current form and can propagate parallelly through all synapses to the post-neurons. From the perspective of the post-neuron, all the currents from the connected synapses are accumulated by Kirchhoff’s law, and the neuron fires output signals based on the nonlinear activation function for consecutive propagation in multilayer neural networks as follows.

(1) $$X^{l+1}=f\left(W^{l}X^{l}\right)$$

where W^*l*^ represents the weight matrix in the l*th* layer, and X^*l*^ is a vector of neuron activations that is applied to the rows of the crossbar array; f() is a nonlinear activation function of the neuron. Thus, a crossbar array that stores multiple bit weights in each RRAM accurately computes the analog-based VMM in a single step. When the inputs are applied to the first neuron layer, the final layer’s output determines the winner neuron after the forward propagation, as shown in Figure 1A. To reduce classification errors between the desired and computed outputs, the calculated errors propagate backward, adjusting each weight to minimize the energy function by gradient descent of the backpropagation algorithm (Figure 1B).

(2) $$\delta^{l}=f^{\prime }\left(W^{l-1}X^{l-1}\right)*{\left(W^{l}\right)}^{T}\delta^{l+1}$$

(3) $$\Delta W^{l}=-\eta \cdot \delta^{l}\cdot {\left(X^{l}\right)}^{T}$$

where δ^*l*^ is the backpropagating error vector of the 1*th* neuron layer and η is the learning rate parameter. * denotes element-wise product. The amount of weight updates in the l*th* layer, Δ*W*^*l*^, becomes the outer product of the two vectors. Therefore, for synaptic devices, the conductance states’ high precision is critical to ensure optimum neural network convergence by adjusting the weights precisely.

### Parallel Update Scheme

When the weights are updated element-wise or row-wise in the crossbar array, the time complexity proportionally increases with an increase in the array size. Crossbar-compatible and fully parallel update schemes have thus been proposed to accelerate neural network training (Burr et al., 2015; Gao et al., 2015; Kadetotad et al., 2015; Gokmen and Vlasov, 2016; Xiao et al., 2020). For the target crossbar array, by applying update pulses simultaneously to all rows and columns based on the neuron’s local knowledge of X and δ, respectively, the parallel updates in each cross point can be executed by the number of pulse overlaps. Therefore, the outer product updates in Eq. (3) are conducted in parallel, as shown in Figure 1B. The pulse encoding method can be implemented in various ways, such as the temporal length, voltage amplitude, and repetition rate. Also, as the update rules can be flexibly adjusted to each system, a parallel updating scheme has been demonstrated in unidirectional phase-change memory (PRAM) arrays (Burr et al., 2015). Therefore, it is vital to employ parallel updating schemes to accelerate neural network training.

## RRAM-Based Hybrid Synapse

This section explains the concept of a hybrid synapse using the RRAMs and their training method to significantly improve the weight resolution and training efficiency of a neural network even with device imperfections. Here, each device that makes up a hybrid synapse is assumed to be implemented in a different array to increase the crossbar’s controllability (Zhu et al., 2019).

### Hybrid Synapse

**FIGURE 2:**
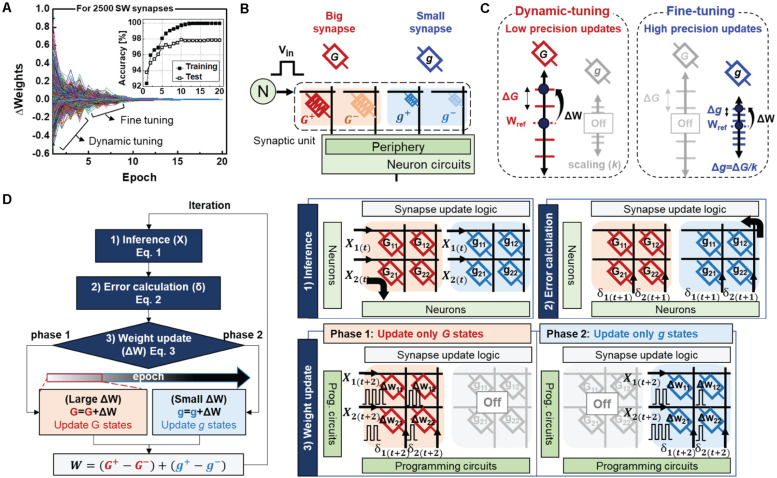
**(A)** Weight changes of ideal synapses as a function of epoch during software training. The inset shows training and test accuracy **(B)** A hybrid synapse is implemented with four different RRAMs. Each RRAM **(C)** Weight states of the big and small synapse for the dynamic-tuning and fine-tuning phases. **(D)** Flow chart and corresponding working principles of the proposed neural network. Here, the subscript (t) indicates the time index of the sequence of actions.

To investigate the ideal synapse behaviors, we first analyzed the weight changes during the software neural network training. Figure 2A shows the weight changes in all synapses in the hidden-output layer as a function of the training epoch. The weight tuning of the software synapses can be mainly divided into two phases: the dynamic-tuning phase, where the weights are largely updated, and fine-tuning phase, where the weights are slightly updated with high precision. Such tendencies are also observed in the training accuracy, which increases rapidly at the initial stage and is then gradually adjusted to the optimum condition, as shown in the inset of Figure 2A. Inspired by this progressive weight update, we present a hybrid synaptic unit with an additional small synapse $\left(g_{\text{ij}}^{+}-g_{\text{ij}}^{-}\right)$ to finely tune the weights after the dynamic tuning phase in the big synapse (Figures 2B,C). Here, g represents conductance states of the small synapse, scaled by *k* times (*g* = *G*/*k*). The larger the scale factor (*k*), the higher the precision of weights that can be expressed. Hence, four devices with different state precisions serve as a synapse, as follows:

(4) $$W_{ij}=\left(G_{ij}^{+}-G_{ij}^{-}\right)+\left(g_{ij}^{+}-g_{ij}^{-}\right)$$

where *G* and *g* represent the conductance states of the low- and high-precision devices, respectively.

### Learning Method

Figure 2D shows a flow chart and the working principle of the proposed neural network. The learning method is mainly composed of three cycles: inference, error calculation, and weight update. During the forward and backward propagations, all big- and small synapses are used to perform VMM operations. In contrast, weight updates are conducted only with specific synapses depending on the training phase. Initially, training starts from the dynamic tuning phase, which only updates the big synapses by switching off the small synapse arrays. Thus, update pulse vectors of X_(*t* + 2)_ and δ_(*t* + 2)_ corresponding to the neuron’s local knowledge of X_(*t*)_ and δ_(*t* + 1)_ are applied to each row and column of the big synapse array, respectively. As the training proceeds, the increase in accuracy may saturate owing to the limited weight resolution of a single synapse. If the accuracy improvement between epochs is below a certain threshold value (the value of 0.5 is adopted for this operation), the update target is switched to a small synapse. Hence, the small synapse’s higher conductance granularity enables finer weight adjustments while the big synapse’s weights are fixed. Therefore, a hybrid synapse with the proposed learning method can overcome the physical limitations of an individual device and accelerate neural network training with only simple switching logic.

### Mo/TiO_x_-Based RRAM

To implement the hybrid synapse, the scale factor *k* can be realized in various ways by scaling the input voltage signal or adjusting the peripheral circuit’s gain. In this work, however, we exploit the switching mechanism of the Mo/TiO_x_-based RRAM, i.e., area-dependent conductance scaling, to implement the gain at the device level. Previously, we reported a microstructural engineered Mo/TiO_x_ RRAM for electronic synapse applications (Park et al., 2019); the study presented some promising synaptic features of the Mo/TiO_x_ RRAM, such as gradual and linear conductance programming. However, the present expanded work adds significantly more explanatory details regarding the areal dependency of the conductance precision, which is utilized to construct a hybrid synapse.

**FIGURE 3:**
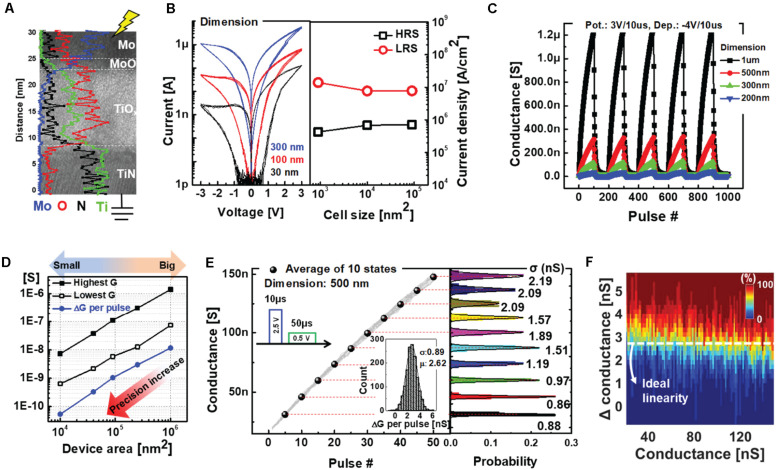
**(A)** Transmission electron microscopy (TEM) image showing the cross section of the Mo/TiO_x_-based RRAM. EDS line profiles are indicated with different colors depending on the materials. **(B)** DC I-V curve and corresponding current density at 0.5 readout voltages of devices with different dimensions. **(C)** AC pulse responses of RRAMs with different dimensions. **(D)** Conductance scaling as a function of active device area. **(E)** Gradual and linear potentiation characteristics of the Mo/TiO_x_-based RRAM for 30 cycling operations. A pulse-train that consists of five –4 V/100 μs pulses is used for strong depression process. **(F)** Heat map showing the cumulative probability to achieve a particular conductance change as a function of the total conductance.

The TiO_x_-based RRAM was fabricated on TiN bottom electrodes with various active diameters from 30 nm to 1 μm. First, we deposited a 15 nm thick TiO_x_ layer through RF sputtering process by using a ceramic Ti_4_O_7_ target at room temperature. Then, 50 nm thick Mo top electrode was deposited by the sputtering system (Park et al., 2019). The device structure and composition of each layer are shown in Figure 3A via transmission electron microscopy (TEM) image and its energy dispersive X-ray spectroscopy (EDS) line profile. The switching mechanism of the RRAM is based on gradual oxygen migration and chemical reactions at the interface between the Mo top electrode and the TiO_x_ layer under an electric field (Park et al., 2019). As shown in Figure 3B, the areal conduction contributes to a gradual increase (or decrease) in the conductance states when a positive (or negative) bias is applied, which are called potentiation and depression, respectively. In Figure 3B, 30 mV step voltage was used for the DC I-V sweep measurement such that 100 sampling points in a single sweep from 0 to 3 voltages. The uniform current density, regardless of device dimensions, demonstrates the interfacial switching of the RRAM.

We also confirmed the areal conduction of the Mo/TiO_x_ RRAM using AC pulse measurements. Figure 3C shows five cycling operations for devices with different dimensions, with 100 pulses each for the potentiation and depression processes. Interestingly, as the effective switching area is scaled down, the entire conductance range of the device decreases proportionally. As shown in Figure 3D, the precision in conductance changes per pulse proportionally increases with device scaling, even with an identical operating scheme. Hence, without modifying the operational scheme, high-precision weights can be represented by scaling the device area k times. For example, when the value of k is 10, the small synapse device area is scaled down by a factor of 10 compared to the big synapse. Following the optimization of the operating scheme, we obtained near-ideal programming linearity during 30 cycles; each cycle included 50 potentiation and one reset process, as shown in Figure 3E. Here, we used the linear potentiation process with a strong reset to maximize the online training accuracy, as a pair device with an occasional reset process allows implementation of the depression as well as negative weights (Burr et al., 2015). The probability distribution shows the excellent state uniformities of 10 representative states, whereas the inset shows the programming variability (δ/μ) with standard deviation (δ) and mean (μ) values. In Figure 3F, the heat map shows the cumulative probability of achieving a particular conductance change as a function of the total conductance to demonstrate linear conductance programming. However, a finite number of conductance states (i.e., 50) in a single device cannot accomplish accurate neural network training comparable to floating-point (FP) implementations. In the next section, we demonstrate the improved training accuracy of the proposed learning method using Mo/TiO_x_ RRAM-based hybrid synapse through neural network simulations.

## Results and Discussion

Simulations were conducted on fully connected neural networks (784-250-10) for pattern recognition of handwritten digits using the Modified National Institute of Standards and Technology (MNIST) dataset. We used 60,000 training and 10,000 test images for the simulations. Also, the mini-batch size was one, and the learning rate was 0.1.

### Simulation Analysis

**FIGURE 4:**
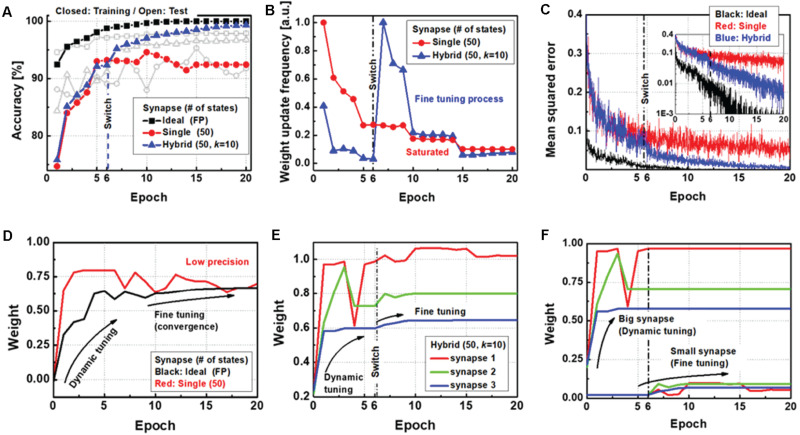
**(A)** Evolution of training and test accuracies, **(B)** weight update frequency response, and **(C)** mean squared error (MSE) of the neural networks as a function of the training epoch for each type of synapse implementation. **(D)** Weight history of the ideal synapse shows dynamic tuning followed by fine tuning with precise adjustments, whereas a single synapse with a low precision device cannot be precisely adjusted. **(E)** Weight history of the hybrid synapse as a function of training epoch. **(F)** Separated weight history for each precision synapse in a hybrid synaptic unit.

As shown in Figure 4A, the performance of the proposed neural network is compared with other types of synapse implementations. First, a single synapse with 50 intermediate states of resistive devices is used to show the saturation of the training accuracy. Although the device has good programming linearity, the finite number of states hinders convergence of the entire network to the optimum condition. However, the software network with FP synaptic weights gradually increases up to 99.98% training accuracy, with 97.92% test accuracy. For the proposed method, the results show 99.66% training accuracy and 97.00% test accuracy, even with the device imperfections. Importantly, unlike the case before switching, where the accuracy remains the same as that of the single-synapse implementation, the accuracy after switching improves gradually. To observe the collaboration of big and small synapses, Figure 4B shows the normalized weight update frequency as a function of the epoch. While the update frequency of the single synapse consistently decreases, the update frequency of the proposed synapse abruptly increases when the target synapse is switched to a small synapse. Then, the number of weight updates decrease again as the synaptic weights are adjusted with high precision during the fine-tuning phase. The convergence of the mean squared error (MSE) of the neural network is analyzed as shown in Figure 4C. After the target update synapse is changed to a small synapse, the stopped MSE reduction starts decreasing gradually.

Figure 4D shows the weight history of a single synapse implementation with 50 states of electronic devices and software synapse implementations with FP weights. In contrast to a single synapse with a finite number of states, the FP synapse converges to its optimum state through the fine-tuning process. Figure 4E shows the case of the hybrid synapse for three representatives. In addition, Figure 4F shows the weight history of the behavior of each low-precision and small synapse. It is seen that dynamic weight tuning is conducted on only the big synapses before switching, whereas fine tuning is conducted on only the small synapses after switching. The results thus demonstrate the successful performance of the proposed method using only 50 intermediate states for each device and a simple array selection logic for the update process.

### Scale Factor (k)

**FIGURE 5:**
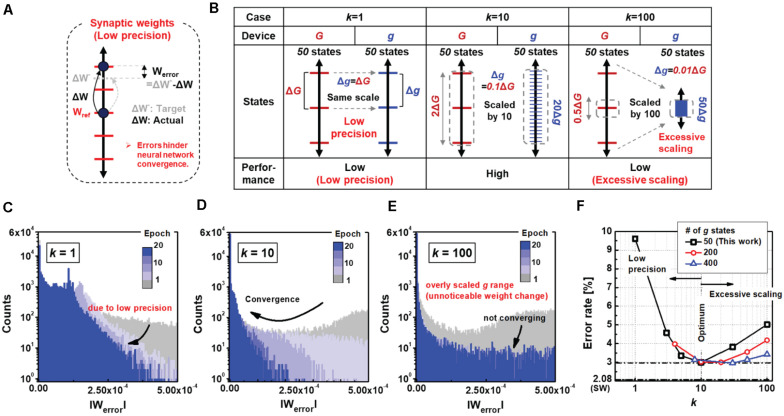
**(A)** Weight states of a single synapse with electronic devices that have finite number of conductance states. During the weight update process, errors may occur due to the low precision. *W*_*error*_ denotes the difference between the target and actual weights. **(B)** Comparison with other cases for *k* values of 1, 10, and 100; a moderate *k* value of 10 is suitable for low errors in weight updates. **(C–E)** Histograms of mean weight errors for different k values. When *k* is 1 **(C)**, the weight resolution does not increase causing low error convergence. When *k* is 100 **(E)**, the weights of small synapses are excessively scaled. Thereby, the overly scaled conductance leads to unnoticeable weight changes with saturated error convergence. **(F)** Classification of error rate as a function of *k* value. At least 10 must be secured to achieve high accuracy with high-precision synaptic weights. Note that saturated error convergence due to a high value of *k* can be improved with a larger number of *g* states in the device for the small synapse.

The gain of the small synapse plays an important role in determining the performance of the neural network, which controls the granularity of the synaptic update. To analyze the optimal value of *k*, we evaluated the errors in the weight updates for different *k* values. As shown in Figure 5A, a synapse with a limited number of states cannot be adjusted to the exact target weight, resulting in weight errors (*W*_error_). Figure 5B shows the precision of the *g* states relative to those of the *G* states for three different cases (*k* = 1, 10, and 100). When *k* is 1, the precision of *g* is as low as that of *G*. If *k* increases to 10, the precision of *g* increases proportionally by 10 times the precision of *G*, such that each state of *G* can be expressed as 10 states of *g*. As a result, after completion of the big synapse training in the proposed neural network, the small synapse can be tuned precisely more than 10 times, thereby further reducing *W*_error_. However, when *k* increases to 100, the weight changes may become unnoticeable owing to the excessively scaled precisions of the *g* states. The 50 finite states of *g* can only express as little as half of the *G* states. Figures 5C–E are histograms for different values of the scale factor showing the distribution of absolute W_error_ values for all synapses in the hidden output layer. As seen in Figure 5D, *W*_error_ gradually decreases when *k* is a moderate value of 10 compared to *k* values of 1 and 100. A low *k* cannot reduce *W*_error_ owing to the low precision of the *g* states (Figure 5C), while an extremely high *k* renders the weight updates of the *g* states unnoticeable (Figure 5E). Therefore, a moderate gain value is important for accurate online training of the network. Figure 5F summarizes the results showing the error rates of the neural networks as functions of *k*. Notably, the increase in error rate due to excessive scaling of *k* can be reduced by a higher number of *g* states. It is seen that the error increases to 5.02% at 100 *k* and decreases to 3.42% when the number of conductance states of the high-precision device increase to 400.

### Performance

**FIGURE 6:**
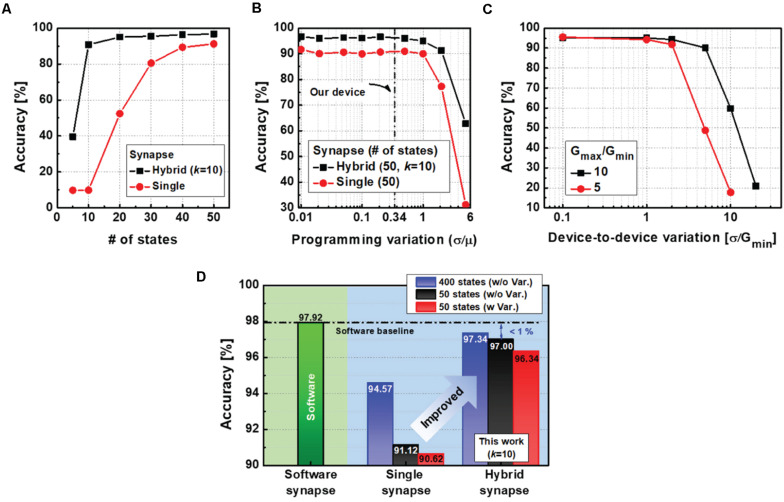
**(A)** Test accuracy as a function of the number of states in an electronic device for each synapse implementation. The hybrid synapse can further improve accuracy even with a finite number of states in a device. **(B)** Test accuracies concerning variations in conductance changes. The proposed neural network with a hybrid synapse has good resistance to variations and enough margin for the variations of our device. **(C)** Test accuracies with various device-to-device variations. **(D)** With the proposed hybrid scheme, neural networks can yield a high accuracy of 97% with only 50 states of the electronic device. When the number of conductance states in the device increases to 400, the neural network accuracy can be improved up to 97.34%.

In addition to analysis of the optimal *k*, we investigated the performance of the neural network reflecting programming variations of the electronic device as well as the number of conductance states. As can be seen from Figure 6A, the hybrid neural network achieves an accuracy of over 93.69% even when the device’s conductance levels are reduced to 10. However, the neural network with single-synapse implementation shows a dramatic decrease to 9.8%. Therefore, the proposed hybrid synaptic unit remarkably reduces the required number of states in the electronic device to obtain the target accuracy.

Moreover, we simulated the impact of programming variations (δ/μ) on the neural network performance for each synapse implementation (Figure 6B). The amount of conductance change (ΔG) is unpredictable, as shown in Figure 3E. To represent the variation of ΔG during the weight update process, we modeled the programming variability by using the mean (μ) and standard deviation (δ) of the ΔG. In the simulation, the variation is assumed to be a random variable with a Gaussian distribution and added to the ΔG in each device during updates. Therefore, we can evaluate the impact of the conductance variation on classification accuracy. Based on the experimental data, variations in our device (0.34) are indicated by the dotted line, where high accuracy of the neural network is still guaranteed. The results show that the neural networks have variational immunity up to one and significantly decrease for variations greater than one. In addition to the programming variation, mean G values can also vary between RRAM devices. In this case, the mean values of large synapses differ so much that there is no overlap between the synapses. Consequently, there would be a limit to the complementary actions between the synapses, leading to severe accuracy loss. Thus, device-to-device variations can play an important role. We have evaluated the impact of device-to-device variations on recognition accuracy in Figure 6C. The network is robust against the variation (δ/*G*_min_) with up to one. It is worth noting that a large ratio of *G*_*max*_/*G*_min_ is essential to improve the immunity to device-to-device variability.

The performances of the different neural networks are summarized in Figure 6D. Compared to the single-synapse implementations with imperfect devices, the hybrid neural networks with the proposed learning method achieve online training accuracies of 97%, which are comparable to FP synapse implementations (97.92%). In particular, the highest accuracy of 97.34% can be achieved when the number of *g* states increases to 400.

### Hybrid Synapse for Spiking Neural Networks

We further discuss how the hybrid synapse and learning method that we proposed can be extended to spiking neural networks (SNNs). Same as the multilayer perceptron model, SNNs can also benefit from dense crossbar array using nanoelectronics devices (Prezioso et al., 2018). The SNN operates by data-driven event-based activations, which makes it promising for energy-efficient neuromorphic hardware. In particular, RRAM has been regarded as a strong candidate with the advantages of high scalability and low power operation, showing spike-timing dependent plasticity (STDP) functionality (Lashkare et al., 2017). Recently, several groups have reported SNNs utilizing multiple devices as a single synapse to secure a higher multi-level conductance state (Werner et al., 2016; Shukla et al., 2018; Valentian et al., 2019).

Meanwhile, SNN has suffered from poor learning performance due to the lack of adequate training algorithms. Many efforts have been made to apply the gradient descent-based backpropagation algorithm to the SNN’s learning to compensate for this issue (Lee et al., 2016). Also, using analog resistive devices, on-chip training SNNs with backpropagation algorithms has been recently reported (Kwon et al., 2020).

 Although the SNN model was not covered in this paper, the proposed multi-element-based synapse and backpropagation-based learning methods are the parts that have been studied in SNN application as well. Our work, therefore, strongly encourages studies on online trainable, fast, and high-accuracy SNN hardware with RRAM synapses.

## Conclusion

To achieve accurate and fast HNN training using RRAM devices, we presented hybrid synaptic unit and the learning method. The hybrid synapse consists of two synapses with different gains; one for dynamic-tuning by large quantities and the other for fine-tuning in detail. By only updating a specific synapse in the synaptic unit depending on the training phase, the weight update process is simplified and we can accelerate the HNN training with a multi-RRAM synaptic architecture. Moreover, we exploited Mo/TiO_x_ RRAM to experimentally demonstrate the hybrid synapse, implementing internal gain at the device level with proportionally scaled areas. Therefore, the granularity of the synaptic weights significantly increased even with the finite number of conductance states in the device. Through neural network simulations, we confirmed it could achieve the highest accuracy of 97.00%, comparable to FP synapse implementations. Finally, we summarized performances with different device parameters by varying the number of states, programming variabilities. We expect this work contributes to building competitive neuromorphic hardware by using RRAM synapses even with the device’s physical limitations.

## Data Availability Statement

The original contributions presented in the study are included in the article/supplementary material, further inquiries can be directed to the corresponding author/s.

## Author Contributions

All authors listed have made a substantial, direct and intellectual contribution to the work, and approved it for publication.

## Conflict of Interest

The authors declare that the research was conducted in the absence of any commercial or financial relationships that could be construed as a potential conflict of interest.
